# Conserved Functional Motifs and Homology Modeling to Predict Hidden Moonlighting Functional Sites

**DOI:** 10.3389/fbioe.2015.00082

**Published:** 2015-06-09

**Authors:** Aloysius Wong, Chris Gehring, Helen R. Irving

**Affiliations:** ^1^Division of Biological and Environmental Science and Engineering, King Abdullah University of Science and Technology, Thuwal, Saudi Arabia; ^2^Monash Institute of Pharmaceutical Sciences, Monash University, Melbourne, VIC, Australia

**Keywords:** moonlighting functional centers, guanylyl/adenylyl cyclase, H-NOX, search motifs, homology modeling, molecular docking

## Abstract

Moonlighting functional centers within proteins can provide them with hitherto unrecognized functions. Here, we review how hidden moonlighting functional centers, which we define as binding sites that have catalytic activity or regulate protein function in a novel manner, can be identified using targeted bioinformatic searches. Functional motifs used in such searches include amino acid residues that are conserved across species and many of which have been assigned functional roles based on experimental evidence. Molecules that were identified in this manner seeking cyclic mononucleotide cyclases in plants are used as examples. The strength of this computational approach is enhanced when good homology models can be developed to test the functionality of the predicted centers *in silico*, which, in turn, increases confidence in the ability of the identified candidates to perform the predicted functions. Computational characterization of moonlighting functional centers is not diagnostic for catalysis but serves as a rapid screening method, and highlights testable targets from a potentially large pool of candidates for subsequent *in vitro* and *in vivo* experiments required to confirm the functionality of the predicted moonlighting centers.

## Introduction

Regulation of proteins is key to cellular function and research has focused on identifying regulatory protein domains and motifs both experimentally and since the advances in -omic databases computationally. Identification of relatively large protein domain signatures (~100 residues in length) by computational methods is achieved with high levels of confidence and is valuable in developing hypotheses about function of novel proteins. However, it is more difficult to predict regulatory motifs, whose identities may be masked by the presence of larger primary domains and thus cannot be identified by regular BLAST-related searches. Motifs critical to binding and catalysis have been characterized on experimental evidence examining molecular binding or enzyme activity using mutational and structural approaches. The three dimensional (3-D) shape of binding sites depends on the folding of the linear sequence of the protein so that the immediate linear motif at the binding site in conjunction with important residues from upstream or downstream of the linear sequence form the critical contact points. Prediction of such 3-D conformation obtained either directly from the amino acid sequence [COMBOSA3D (Stothard, 2001); motif3D (Gaulton and Attwood, 2003)] or from the protein structures [PROMOTIF (Hutchinson and Thornton, 1996), MotAn (Aksianov, 2014)] can infer structural and/or functional information, for example, binding to organic molecules, co-factors, DNA/RNA, and other interacting protein partners. Further, PDBeMotif (Golovin and Henrick, 2008) allows the prediction of modifications resulting from the binding to small molecules at the catalytic and/or regulatory sites based on sequence, chemical, and structural analysis across the PDB database.

Multiple computational approaches now exist to seek motifs that identify binding and/or active sites in proteins as well as key determinants for protein substrate sites. These approaches include detection of post-translational modification sites including phosphorylation sites such as PhosphoSite (Hornbeck et al., 2004) or glycosylation sites such as glycosylation predictor (Hamby and Hirst, 2008). Several investigators have developed sites focused on predicting short linear motifs that can act as regulatory points in part because they can be post-translationally modified. These sites include DILIMOT (Neduva and Russell, 2006), SLiMSearch (Davey et al., 2011); ELM (Puntervoll et al., 2003; Gould et al., 2010; Dinkel et al., 2014), and MiniMotif (Mi et al., 2012). The switches.ELM Resource is a curated resource of experimentally identified short linear motifs that are pre- or post-translationally modified and are predicted to act as molecular switches (Van Roey et al., 2013). Additional computational approaches exist such as CAPRI (Lensink and Wodak, 2010) and MDockPP (Huang and Zou, 2010) that predict protein–protein docking; ITScore-PR, which uses an iterative method based on experimentally determined RNA–protein complex interactions (Huang and Zou, 2014); and RPI-Pred that predicts protein–RNA interactions based on sequence and structural information (Suresh et al., 2015). All such approaches provide predictions and it is important to undertake appropriate measures to avoid false positives as discussed in detail in previous studies (Iyer et al., 2001; Gould et al., 2010; Mi et al., 2012; Gibson et al., 2013; Dinkel et al., 2014). For instance, the Minimotif Miner 3.0 includes false-positive filters and scoring to assist users in avoiding false positives (Mi et al., 2012).

Protein surfaces are relatively large and there is potential for multiple interactions with small ligands and other protein(s). Some interactions may result in allosteric modification of the originally defined function of the protein whereas others may reveal a new function. These later sites we term hidden moonlighting functional centers as they have only recently begun to be characterized but do not fit the original description of moonlighting proteins (Jeffery, 2009, 2014). In this article, we review how hidden moonlighting functional centers in proteins can be identified using targeted bioinformatic searches predominantly combining carefully curated functional search motifs with homology models and docking evaluations. Hidden moonlighting functional centers can be binding sites with catalytic activity or they may be binding sites that regulate protein function in a novel manner. We use as our examples, molecules that were identified via bioinformatics searches initially seeking cyclic mononucleotide cyclases in plants.

## A Motif-Based Search for Nucleotide Cyclases in Higher Plants

Cyclic mononucleotides have important and diverse physiological roles in signaling in higher plants. These roles include the activation of cyclic nucleotide-responsive protein kinases, the interaction with cyclic nucleotide-binding proteins, and the gating of cyclic nucleotide-gated ion channels (Newton and Smith, 2004; Meier and Gehring, 2006; Zelman et al., 2012), and it is therefore highly unlikely that a single adenylyl cyclase (AC) or guanylyl cyclase (GC) in higher plants could account for all cAMP- and cGMP-dependent processes reported to date. This leaves us with the task of identifying candidate nucleotide cyclases (NCs) in higher plants, a task that is further complicated by the fact that BLAST searches including ancillary pattern-hit initiated- (phi-), position-iterated- (psi-), and domain enhanced lookup time accelerated (delta-) BLAST with annotated ACs or GCs from prokaryotes and lower and higher eukaryotes did not yield plausible candidates (Ludidi and Gehring, 2003; Gehring, 2010).

**Figure 1:**
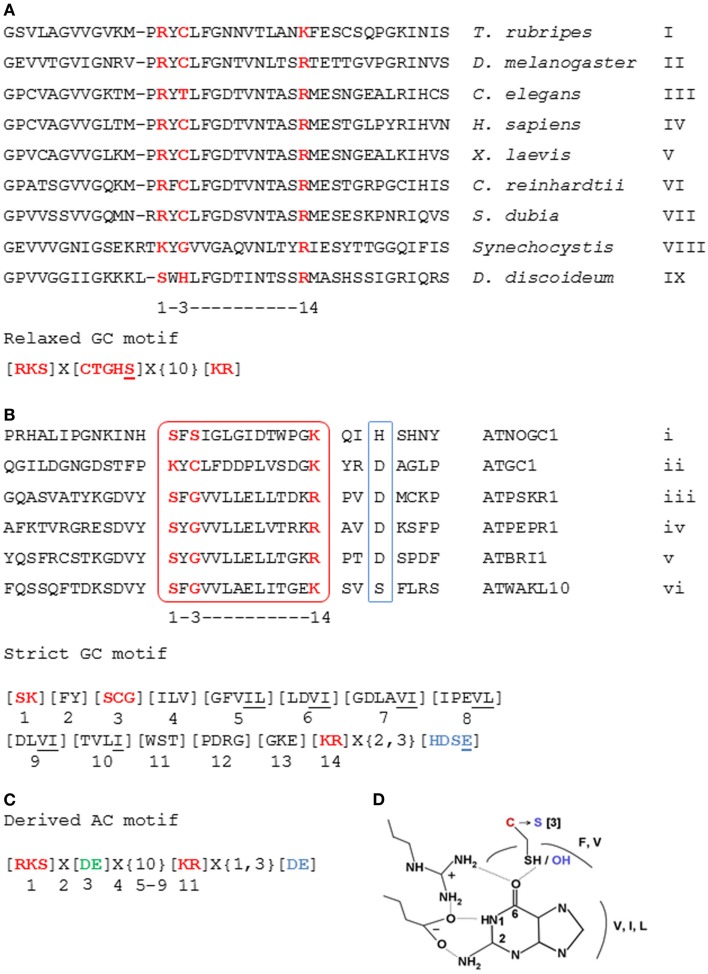
**Alignment of the sequences of GC catalytic centers and construction of NC search motifs**. **(A)** The 14 amino acid long “relaxed” GC search motif deduced from the alignment of GC catalytic domains across species and **(B)** the “strict” GC search motif deduced from the alignment of catalytic centers (boxed in red) of previously characterized plant GCs. **(C)** The AC motif derived from the “relaxed” GC motif by substitution of the residue at position 3 with “D” or “E” (highlighted in bold green) to confer specificity to ATP. **(D)** The substitution of “C” at position 3 of the GC motif to “S” converts a thioester into an ester. The interacting residues having similar chemical properties to those present in the respective positions of the motif are also indicated. Accession numbers of aligned sequences are as follows: I, NP_001027855; II, NP_524603; III, NP_494995; IV, NP_000171; V, BAA83786; VI, BI717053; VII, AL132834; VIII, NP_440289; IX, CAB42641; i, NP_176446; ii, NP_568159; iii, NP_178330; iv, NP_177451; v, NP_195650; and vi, NP_178086. The amino acid substitutions are in square brackets ([]); “X” stands for any amino acid; and the gap size is marked in curly brackets ({}). Underlined amino acids are residues added to the motif due of their chemical similarity to the amino acid normally found in this position. Amino acids in red are functionally assigned residues, and those in blue (or boxed in blue) are implicated in binding with Mg^2+^ or Mn^2+^ ions. Figures were modified from Ludidi and Gehring (2003), Gehring (2010), and Wong and Gehring (2013).

It was, however, hypothesized (Garbers and Lowe, 1994) that plant GCs might contain a significant degree of amino acid sequence conservation and structural similarity in the catalytic center to previously identified NCs and notably natriuretic peptide receptors, some of which are known to signal via cGMP (Chinkers et al., 1989; Garbers and Lowe, 1994; Wedel and Garbers, 1997). If so, it could be expected that the residues directly implicated in catalysis (Liu et al., 1997) would show a high degree of conservation. Alignments of such catalytic centers of annotated GCs from vertebrates, lower eukaryotes, and prokaryotes allowed the building of a 14 amino acid long search motif that includes amino acid residues at positions 1, 3, and 14, which have been assigned functions that are important for catalysis (Figure 1A). The amino acid in position 1 binds to guanine of GTP; the residue in position 3 confers substrate specificity discriminating GTP from ATP; and the amino acid in position 14 binds to the phosphate acyl group and stabilizes the transition of GTP to cGMP. This GC motif has led to the identification of the first candidate GC (i.e., ATGC1) in higher plants that showed catalytic activity *in vitro* (Ludidi and Gehring, 2003) (Figures 1A,B). Later, a related molecule regulated by light was identified in morning glory (*Pharbitis nil*) (Szmidt-Jaworska et al., 2009).

The experimental proof of concept opened the way to the discovery of additional candidate GCs, using rationally modified motifs and testing their functionality both *in vitro* and *in vivo*. These candidates include the ATPSKR1, ATPEPR1, ATBRI1, and ATWAKL10, which have all been shown to harbor functional GC catalytic centers *in vitro* and in the case of ATPSKR1 in isolated protoplasts (Kwezi et al., 2007, 2011; Meier et al., 2010; Qi et al., 2010). Interestingly, these molecules are all examples of receptor-like kinases with functional kinase activity (Clouse, 2011; Hartmann et al., 2014). Further, in ATPSKR1, binding of the natural ligand phytosulfokine-alpha also resulted in elevated amounts of cGMP in isolated mesophyll protoplasts (Kwezi et al., 2011) while recently, the molecular regulation of ATPSKR1 kinase and GC catalytic activities has been elucidated (Muleya et al., 2014).

In order to test if the catalytic functions of the key residues can be rationally performed by other amino acids of similar chemical and/or physical properties that were absent from the first motif, we added a serine residue at position 3 to make the “relaxed” GC motif (Figure 1A). This substitution converts a thioester into an ester configuration (Figure 1D). Indeed, this extension of the search motif has led to the identification of a further functional GC, the ATNOGC1 (Mulaudzi et al., 2011).

Based on the sequences of the six characterized plant GCs, to date, a more stringent GC motif that includes the amino acids present in each position of the 14 amino acid long GC catalytic center was deduced (Figure 1B) (Wong and Gehring, 2013). The rationale for this is to further identify molecules that harbor similar GC catalytic centers based on evidence indicating that these molecules are functional *in vitro* and/or *in vivo*, and to construct a more rigid motif that is “plant-specific”; since they are derived from the alignment of plant molecules. Additionally, the amino acid implicated for metal binding (Mg^2+^ or Mn^2+^) appearing at two or three residues downstream of the catalytic center, as well as the residues that have similar chemical properties to amino acids in the respective positions of the motif were included (Figure 1B). This stringent GC motif identified >40 candidate molecules in *Arabidopsis*, thus implying that in higher plants, there remain a substantial number of undiscovered proteins with potentially functional GC catalytic centers (Wong and Gehring, 2013). Many of these predicted GC catalytic centers share residence with kinase domains thereby constituting a class of multi-functional plant proteins with hidden catalytic centers that could represent a novel group of moonlighting proteins (Irving et al., 2012).

The motif search method that proved useful in the discovery of GC can also be used in the search for candidate ACs (Gehring, 2010). This approach was based on the report that a *Dictyostelium discoideum* homolog of a mammalian soluble AC encodes a GC (Roelofs et al., 2001). Site-directed mutagenesis causing amino acid substitutions in the residues responsible for substrate specificity (Figure 1) can turn an AC into a GC and *vice versa* (Sunahara et al., 1998; Tucker et al., 1998). In the modified AC search motif, the amino acid in position 3 is substituted to “D” or “E” to confer specificity for ATP (Figure 1C) and selected *Arabidopsis* candidates harboring the AC motif (Gehring, 2010) are being investigated.

The motif searches are best done in organisms where the complete sequences are available in the public domain, for example, *Arabidopsis thaliana*. *Arabidopsis* is particularly amenable since a ready search interface (PatMatch) is available on The Arabidopsis Information Resource (TAIR) web page (www.arabidopsis.org) (Yan et al., 2005). Perhaps the biggest problem is the identification of false positives, and to address this, several computational approaches that help build confidence in a prediction can be performed. First, if orthologs of candidate ACs or GCs in related species also have the catalytic center motif, it increases confidence in the prediction, and a convenient way to test this is by using a Pattern-Hit Initiated BLAST (Phi-BLAST). In addition, scouting available databases [for example, TAIR (Huala et al., 2001) and Genevestigator (Zimmermann et al., 2004) for research related to *Arabidopsis*] for information regarding the protein’s cellular localization, solubility, expression levels across tissues, at different growth stages and any changes in response to hormones, chemicals, pathogen, and abiotic stresses can establish relevance between the predicted molecular functions to known cellular and/or biological functions. If candidates appear to potentially fit into the cellular context where the predicted functionality has a role, they can then be examined *in silico*. Second, and particularly if good template structures are available, three dimensional homology structural models of candidate NCs can be made (Wong and Gehring, 2013). These models when combined with substrate docking simulations also allow the assessment of structural changes at the catalytic center and offer a way to do *in silico* site-directed mutagenesis (Wong and Gehring, 2013) to further probe substrate specificity, binding pose, and interactions with key residues at the catalytic center.

## *In silico* Characterization of Plant Nucleotide Cyclase Catalytic Centers

**Figure 2:**
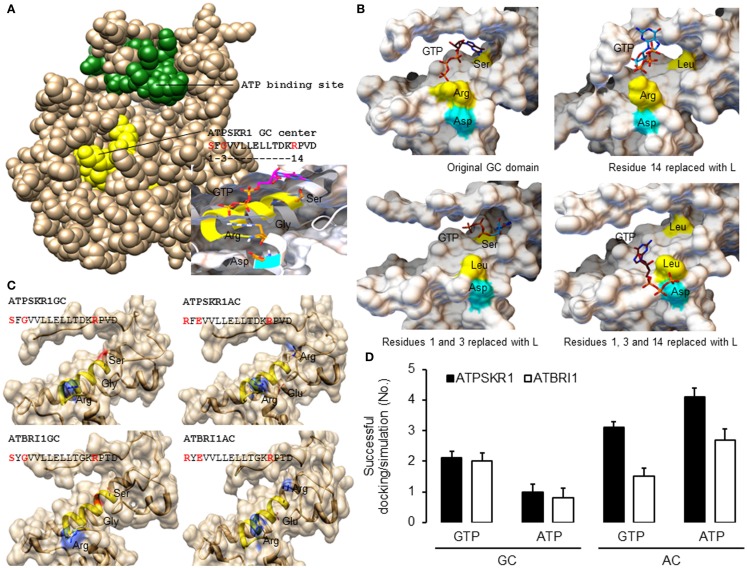
**Homology models of *Arabidopsis* GC catalytic centers and molecular docking of GTP and ATP**. **(A)** The homology model of ATPSKR1 kinase (Phe^734^–Val^1008^) illustrates the domain organization of the ATP binding site (green) and the moonlighting GC center (yellow) and the molecular docking of GTP to the GC center (inset) reveals substrate pose and interactions with key residues at the GC center. Ribbons highlighted in yellow and cyan indicates the GC catalytic center and the metal binding residue. **(B)** Docking simulations of GTP to ATPSKR1 GC catalytic center (Asn^871^–Glu^980^) that has one or more key amino acid residues replaced. Functional amino acid residues at positions 1, 3, and 14 of the motif are indicated in yellow and the residue that is involved in metal binding is highlighted in cyan. The substrate orientation was defined as “suitable for catalysis” if the hydrophobic nucleobase guanine or adenine sits deep at the catalytic center and at distance close enough to establish interactions important for catalysis with the experimentally determined functional residues at positions 1 and 3 of the motif while the negatively charged hydrophilic triphosphates point outwards toward the solvent exposed amino acid residue at position 14 of the motif (arginine or lysine) that has a positive net charge. In addition, this orientation also places the triphosphate end in the direction of interacting co-factors (Mg^2+^ or Mn^2+^) that bind with the amino acid (aspartic acid or glutamic acid) located two residues downstream of the motif. Figures were modified from Wong and Gehring (2013). **(C)** Models of the secondary and tertiary structures of ATPSKR1 (Asn^871^–Glu^980^) and ATBRI1 (Leu^1021^–Arg^1134^) catalytic centers at their native GC and GC-derived AC states. Residues at positions 1 and 3 were replaced with “R” and “E,” respectively, to match the AC motif, turning the GC catalytic centers of ATPSKR1 and ATBRI1 into putative ACs. The GC catalytic center (yellow ribbon) and the key catalytic residues are highlighted accordingly. All structures and images were prepared and analyzed using UCSF Chimera (Pettersen et al., 2004). **(D)** Docking simulations of GTP and ATP on the GC and the putative AC catalytic centers of ATPSKR1 and ATBRI1. A total of 10 docking simulations each were performed, generating nine solutions and the positive binding modes in each run were determined by analysis with PyMOL (ver 1.7.4) (The PyMOL Molecular Graphics System, Schrödinger, LLC), and the number of successful dockings per simulation were averaged. Homology models of ATPSKR1GC (Asn^871^–Glu^980^) and ATBRI1GC (Leu^1021^–Arg^1134^) were based on the AvrPtoB–BAK1 complex (PDB entry: 3TL8) using Modeller (ver. 9.10) (Sali and Blundell, 1993) and NTP docking experiments were performed using AutoDock Vina (ver. 1.1.2) (Trott and Olson, 2010).

In order to generate good quality models, template structures that have high degree of similarity to the queried amino acid sequence especially at regions of interest should be selected for homology modeling. Here, the kinase domain of ATPSKR1 (Phe^734^–Val^1008^) was modeled against the AvrPtoB–BAK1 complex (PDB entry: 3TL8), which has a sequence similarity of 43% covering 99% of the queried amino acid sequence. Although the GC center of ATPSKR1 is located within a larger kinase domain the GC catalytic center does not overlap with the ATP binding site of the kinase (Figure 2A) thus suggesting that both centers can perform catalysis independently and may be concurrently active. However, molecular conditions that favor the activation of one can invoke structural alterations that impede the activity of the other. For example, *in vitro* studies with ATPSKR1 showed that Ca^2+^ was the molecular switch that selectively activates the moonlighting GC activity and inhibits the primary kinase (Muleya et al., 2014). To obtain structural insights on the predicted GC catalytic centers, a narrower region that only accommodates the GC centers was modeled against the AvrPtoB–BAK1 complex (PDB entry: 3TL8) since in kinase active configuration, the GC centers appear to be partially buried (Figure 2A). In addition to specific molecular conditions, dimerization events can also lead to an “open” GC center and this has been discussed elsewhere (Wong and Gehring, 2013). The predicted GC-specific structures can then be subjected to molecular docking simulations, which allow specific probing of substrate binding, orientation, and interactions with the key residues of the catalytic centers. In general, the homology models of all characterized plant GCs have common features at the catalytic center, i.e., an alpha helix fold that accommodates the majority of the residues including those at positions 1 and 3 of the motif, and is followed immediately by a loop that contains the residue at position 14 of the motif. The key residues, in particular, the amino acids at positions 1 and 14 are positioned favorably and are free to interact with the guanine and phosphate ends of the docked GTP (Figure 2A). Notably, docking simulations suggest that GTP docked with a good free-energy and favorable binding mode, i.e., GTP was positioned in an orientation deemed suitable for catalysis (Figure 2A). At the tertiary level, all the models have a distinct cavity that can rationally fit the GTP or ATP substrate, with the amino acid residue at position 1 of the motif sitting deep within the hydrophobic core and the residue at position 14 occupying the opening of the cavity (Figure 2B). The predicted function, in particular, the substrate binding role of key residues at the catalytic center was investigated by site-directed mutagenesis of the models. These residues were systematically replaced with leucine, and docking simulations were run using models incorporating these mutations. In the evaluation of the ATPSKR1GC representative model, GTP was predicted to either fail to dock or docked with orientation deemed unsuitable for catalysis as previously defined at the catalytic center of structures that have the mutations (Figure 2B). These predictions suggest that these residues are implicated in binding of GTP at the catalytic center, and substrate binding is a required step that precedes the conversion of GTP to cGMP.

We further mutated the amino acid residues at positions 1 and 3 to “R” and “E,” respectively, to convert the GC into a putative AC. In addition to the residue that confers substrate specificity at position 3, we also changed the amino acid at position 1 of the motif, replacing “S” with “R” since “R” appears only in the AC motif (Figure 1C) and not in the “strict” GC motif (Figure 1B). In both representative ATPSKR1GC and ATBRI1GC structures, these mutations did not drastically alter the shape of the catalytic center, although the surface charge and the hydrophobic environment of the cavity were affected (Figure 2C). Indeed, docking simulations suggest that the putative GC-derived AC domains of ATPSKR1 and ATBRI1 now bind ATP at higher probability than GTP (Figure 2D) and this agrees with previous studies showing ACs and GCs to have interchangeable catalytic functions governed by the residue conferring substrate specificity (Roelofs et al., 2001). A recent study on the effects of cationic residues on the hydrophobic interactions indicates that arginine weakens hydrophobic interactions whereas lysine strengthens them in proteins in general (Ma et al., 2015) and further supports the need to include “R” at position 1 in our models.

Typically, ACs and GCs are complex signaling molecules that contain other domains, notably kinase domains, and come in many different domain combinations and architectures (Meier et al., 2007; Biswas et al., 2009) that rely on structural malleability to perform their primary and any moonlighting functions (Tompa et al., 2005). Central to the regulation of these multi-domain proteins are their interactions with molecular switches such as Ca^2+^ binding (Muleya et al., 2014) and the formation of homo-dimers (Misono et al., 2011). Absence of such conditions, especially *in vitro*, is one likely reason for the apparent absence of GC activity in the BRI1 kinase reported previously (Bojar et al., 2014) and discussed elsewhere (Freihat et al., 2014). Notably, GC motifs are also discovered in mammalian kinases, in particular, the human interleukin 1 receptor-associated kinase 3 (IRAK3), which has been shown to generate cGMP *in vitro* and as a GFP-fusion protein in (HEK)-293T cells (Freihat et al., 2014). The amount of cGMP generated by human IRAK3 is comparable to that of ATPSKR1 but lower than typical amounts of mammalian GCs. This suggests that rather than a long-distance signaling role, the predicted GCs may have a modulatory role that serve as diversion points in complex signal transduction networks, switching from one pathway to another. This requires only localized cGMP effects such as that afforded by a reduced cGMP level of plant GCs in general as well as human IRAK3 (Freihat et al., 2014).

## Auxiliary Allosteric Regulatory Binding Sites

The fact that ACs and GCs can have multiple domains (Biswas et al., 2009; Misono et al., 2011) and a variety of architectures suggest additional potential allosteric binding sites in plant GCs. These auxiliary binding sites provide regulatory functions that can enable the protein to shift from one signaling pathway to another by switching on or off the activity of the primary or moonlighting functional centers. This feature can be used to perform searches with multiple motifs and has been successfully applied to identify the first nitric oxide (NO) binding GC in plants (Mulaudzi et al., 2011). In this case, a GC catalytic center motif was combined with a derived heme-nitric oxide/oxygen binding (H-NOX) motif (Boon et al., 2005). Specifically, this H-NOX motif Hx[12]Px[14,16]YxSxR was used in tandem with the GC motif (Figure 1A) in a PatMatch search against the *Arabidopsis* proteome that retrieved ATNOGC1, which is not only a functional GC but also has catalytic activity that is dependent on the binding of NO (Mulaudzi et al., 2011; Domingos et al., 2015), thus implying that NO-dependent biological responses may be mediated by cGMP in plants. This finding is particularly relevant to developing hypotheses about the regulation of NO/cGMP signaling pathways in plants, which seems to resemble that in the animal system and, therefore, bridges the link between NO perception and the NO-dependent biological response (Domingos et al., 2015).

## Conclusion

In conclusion, rationally designed search motifs can be used to reveal candidate proteins that harbor moonlighting functional centers, which cannot be identified by BLAST-related searches due to poor residue conservation that is further masked by the presence of larger primary domains. The strength of this computational approach is enhanced when orthologs are also found and good homology models can be developed to test the functionality *in silico*, which provides useful knowledge on events crucial for catalysis such as substrate binding, orientation, and interaction with key residues of the predicted catalytic center. We note that this computational-based characterization of hidden functional centers is not diagnostic for catalysis and therefore does not distinguish enzymes that catalyze the same substrate, for example, the discrimination of GTPases from GCs (Wong and Gehring, 2013). Thus, *in vitro* and *in vivo* experiments are necessary to confirm the functionality of the moonlighting domains identified in this manner. However, motif-based searches coupled with binding simulations can serve as a rapid initial screen to make better informed decisions regarding the selection of probable candidates and avoid the isolation of false positives. The confidence in the prediction of the selected molecules to perform their predicted moonlighting functions is therefore increased, and they can then be brought to the fore from a potentially large pool of candidate proteins.

## Conflict of Interest Statement

The authors declare that the research was conducted in the absence of any commercial or financial relationships that could be construed as a potential conflict of interest.
